# A Feature Extraction Method of Ship-Radiated Noise Based on Fluctuation-Based Dispersion Entropy and Intrinsic Time-Scale Decomposition

**DOI:** 10.3390/e21070693

**Published:** 2019-07-15

**Authors:** Zhaoxi Li, Yaan Li, Kai Zhang

**Affiliations:** 1 School of Marine Science and Technology, Northwestern Polytechnical University, Xi’an 710072, China; 2 Department of Computer & Information of Science & Engineering, University of Florida, Gainesville, FL 32611, USA

**Keywords:** ship-radiated noise, fluctuation-based dispersion entropy (FDispEn), instrinsic time-scale decomposition (ITD), proper rotation component (PRC), feature extraction

## Abstract

To improve the feature extraction of ship-radiated noise in a complex ocean environment, fluctuation-based dispersion entropy is used to extract the features of ten types of ship-radiated noise. Since fluctuation-based dispersion entropy only analyzes the ship-radiated noise signal in single scale and it cannot distinguish different types of ship-radiated noise effectively, a new method of ship-radiated noise feature extraction is proposed based on fluctuation-based dispersion entropy (FDispEn) and intrinsic time-scale decomposition (ITD). Firstly, ten types of ship-radiated noise signals are decomposed into a series of proper rotation components (PRCs) by ITD, and the FDispEn of each PRC is calculated. Then, the correlation between each PRC and the original signal are calculated, and the FDispEn of each PRC is analyzed to select the Max-relative PRC fluctuation-based dispersion entropy as the feature parameter. Finally, by comparing the Max-relative PRC fluctuation-based dispersion entropy of a certain number of the above ten types of ship-radiated noise signals with FDispEn, it is discovered that the Max-relative PRC fluctuation-based dispersion entropy is at the same level for similar ship-radiated noise, but is distinct for different types of ship-radiated noise. The Max-relative PRC fluctuation-based dispersion entropy as the feature vector is sent into the support vector machine (SVM) classifier to classify and recognize ten types of ship-radiated noise. The experimental results demonstrate that the recognition rate of the proposed method reaches 95.8763%. Consequently, the proposed method can effectively achieve the classification of ship-radiated noise.

## 1. Introduction

Ship-radiated noise is an important indicator to measure the performance of a ship. It is generally believed that the ship-radiated noise is superimposed by mechanical noise, propeller noise and hydrodynamic noise. The study of ship-radiated noise feature extraction is of great significance in the identification and classification of underwater passive targets. Therefore, extracting effective and reliable ship-radiated noise characteristic parameters is the focus of research in the field of underwater acoustics. The processing of ship-radiated noise signal is a typical nonlinear, non-Gaussian and non-stationary signal processing problem. Traditional signal processing methods such as short-time Fourier transform, LOFAR spectral analysis, and wavelet transform finds it difficult to accurately extract the characteristics of the ship-radiated noise [1,2]. The Second-Generation Wavelet is no longer dependent on the frequency domain and easily implements fast algorithms. At the same time, it has a good ability to suppress noise components in non-stationary signals. However, it is still affected by the wavelet basis function and decomposition level [3]. 

In recent years, it has been important to preprocess the signal to eliminate noise in the original signal and to reduce the effects of aliasing between the feature information. Commonly used preprocessing methods are: empirical mode decomposition (EMD) [4,5], ensemble empirical mode decomposition [6] (EEMD) and other signal decomposition methods. Li Yuxing [7] used EEMD to analyze the strongest IMF center frequency of ship-radiated noise. Among different categories of ship-radiated noise, by comparing their characteristic parameters from strongest IMF center frequency with ones from high and low frequencies—the same types of ships basically show a similar level of characteristic parameters, while the different types of ships exhibit discrepancies. Intrinsic time-scale decomposition (ITD) is a method that can effectively process nonlinear and non-stationary signals based on EMD and local mean decomposition (LMD) methods. ITD can accurately extract the dynamic characteristics of non-stationary signal with few numbers of iterations and small edge effect such that it is capable of online processing in real time. Over the past decades, it has been widely used in the fault diagnosis [8], biomedicine [9,10,11], geophysics [12], hydroacoustics [13], etc.

The entropy value indicates the complexity of the signal. It can be used to effectively reduce the dimension of the feature vector and fully represent the characteristic of the signal. There are many methods for characterizing the complexity of time series, such as Shannon entropy [14], sample entropy [15], permutation entropy [16,17], etc., which have been successfully applied in the field of fault diagnosis and the medical field. However, sample entropy is time consuming for long data calculations and is susceptible to mutated signals. Although the permutation entropy is faster, it fails to consider the relationship in signal amplitudes. In order to overcome the shortcomings of sample entropy and permutation entropy, a new measure of complexity, the dispersion entropy, was proposed by Mostafa Rostaghi and Hamed Azami in 2016 [18]. The dispersion entropy based on Shannon entropy was developed to quantify the uncertainty of the time series. In [19], the fluctuation of the signal is used to develop fluctuation-based dispersion entropy (FDispEn), which tackles the limitations of permutation entropy and sample entropy. It takes the relationship in amplitudes into account, but also requires modest computations and shows significant robustness.

Based on the theory of ITD and fluctuation-based dispersion entropy, we propose a method combining ITD and fluctuation-based dispersion entropy to the feature extraction of ship-radiated noise. Firstly, the measured different ship-radiated noise is decomposed by ITD, and then the proper rotational components (PRCs) with high correlation are extracted and the value of the fluctuation-based dispersion entropy of each order PRC is calculated. The fluctuation-based dispersion entropy value of the PRC containing the main information is determined as the feature vector. Finally, the feature vector is put into SVM for classification. 

## 2. Methods

### 2.1. Intrinsic Time-Scale Decomposition (ITD)

Intrinsic Time-scale Decomposition (ITD) is a relatively new, nonlinear, non-stationary signal processing method proposed by Frei and Osorio in 2006. It can extract the instantaneous frequency characteristics of the signal more accurately in real time, and decompose the non-stationary signal into a series of proper rotation components (PRCs) containing significant instantaneous frequency component and a residual component. the ITD method is introduced as follows:

Suppose $X_{t}$ is a real-valued discrete signal. Let ξ denote the baseline extraction factor of $X_{t}$, $H_{t}=X_{t}-\xi X_{t}$ denote a proper rotation component, where $\xi X_{t}$ denotes as $L_{t}$, is the mean curve of the signal. Then the ITD algorithm steps are as follows [20]:

(1) Let $X_{k}$ denote the all local extremas of $X_{t}$ at time points $\tau_{k}\left(k=1,2,\cdots ,N\right)$, and define $\tau_{0}=0$. We can define baseline signal point as:
  (1) $$L_{k+1}=\alpha [X_{k}+\left(\frac{\tau_{k+1}-\tau_{k}}{\tau_{k+2}-\tau_{k}}\right)\left(X_{k+2}-X_{k}\right)]+\left(1-\alpha \right)X_{k+1},\left(k=1,2,3,\cdots ,N-2\right)$$
  where α∈[0,1] and is typical selected as 0.5.
(2) We can define piecewise linear baseline extracting operator of the signal $X_{t}$ as:
  (2) $$L_{t}=\xi X_{t}=L_{k}+\left(\frac{L_{k+1}-L_{k}}{X_{k+1}-X_{k}}\right)\left(X_{t}-X_{k}\right)$$
(3) Using the baseline signal $L_{t}$ as the original signal, repeat steps (1–2), and the original signal is decomposed into:
  (3) $$X_{t}=\xi X_{t}+HX_{t}=\left(H\sum_{k=0}^{p-1}\xi^{k}+\xi^{p}\right)X_{t}$$
  where, $H\xi^{k}X_{t}$ is the k+1 proper rotation component (PRC), $\xi^{p}X_{t}$ is monotonic trend signal.

The superiorities of ITD over EMD and other classic signal analysis methods are:

1. ITD eliminates the needs of sophisticated ‘sifting’ and spline interpolation of local extremas, which are necessary in EMD method. Therefore, the reduction of the computational complexity of ITD allows obtaining the instantaneous parameters of the signal in real time.
2. The various instantaneous parameters obtained by ITD decomposition can accurately express the time-varying characteristics of the non-stationary signal frequency (the instantaneous time resolution of the time-frequency information is equal to the time scale corresponding to the extreme point in the input signal). It is worth noting that the time-frequency information is not restricted by the time-frequency uncertainty comparing with one obtained by traditional integral transform.

### 2.2. Fluctuation-Based Dispersion Entropy

Fluctuation-Based Dispersion Entropy (FDispEn) is a nonlinear dynamic analysis method that characterizes the complexity and irregularity of time series. The algorithm is based on the mapping of normal distribution functions. Therefore, the expectation and standard deviation of the data should be considered. The calculation steps of FDispEn are summarized as follows [19]:

(1) Define time series is $x=\left\{x_{j},j=1,2,\cdots ,N\right\}$, x is mapped to $y=\left\{y_{j},j=1,2,\cdots ,N\right\}$, according to normal distribution function, where $y_{i}\in \left(0,1\right)$ and the normal distribution function $y_{i}$ is defined as:
  (4) $$y_{j}=\frac{1}{\sigma \sqrt{2\pi }}\int_{-\infty }^{x_{j}}e^{\frac{-{\left(t-\mu \right)}^{2}}{2\sigma^{2}}}dt$$
  where, μ and σ respectively represents expectation and standard deviation of time series.
(2) The y is linearly mapped to an integer from 1 to c.
  (5) $$z_{j}^{c}=round\left(c\cdot y_{j}+\frac{1}{2}\right)$$
  where $z_{j}^{c}$ shows the j-th member of the classified time series and rounding involves either increasing or decreasing a number to the next digit.
(3) Calculate the embedded vector $z_{i}^{m,c}$:
  (6) $$z_{i}^{m,c}=\left\{z_{i}^{c},z_{i+d}^{c},\cdots ,z_{i+(m-1)d}^{c}\right\},i=1,2,\ldots ,N-\left(m-1\right)d$$
(4) The dispersion pattern is defined as: $\pi_{v_{0},v_{1},\cdots ,v_{m-1}}\left(v=1,2,\cdots ,c\right)$. Each $z_{i}^{m,c}$ is mapped to a dispersion pattern $\pi_{v_{0},v_{1},\cdots ,v_{m-1}}$, where $z_{i}^{c}=v_{0},z_{i+d}^{c}=v_{1},\ldots ,z_{i+(m-1)d}^{c}=v_{m-1}$. The number of possible dispersion patterns assigned to each vector $z_{i}^{m,c}$ is equal to ${\left(2c-1\right)}^{m-1}$ since $z_{i}^{m,c}$ has m elements and each can be one of the integers from −c+1 to c−1.
(5) For each dispersion pattern $\pi_{v_{0},v_{1},\cdots ,v_{m-1}}$, relative frequency $p\left(\pi_{v_{0,}v_{1},\cdots ,v_{m-1}}\right)$ is defined as follows:
  (7) $$p\left(\pi_{v_{0,}v_{1},\cdots ,v_{m-1}}\right)=\frac{N_{b}\left(\pi_{v_{0},v_{1},\cdots ,v_{m-1}}\right)}{N-\left(m-1\right)d}$$
  where, $N_{b}\left(\pi_{v_{0},v_{1}^{},\cdots v_{m-1}}\right)$ represents the number of $z_{i}^{m,c}$ mapped to $\pi_{v_{0},v_{1},\cdots ,v_{m-1}}$. Actually, $p\left(\pi_{v_{0},v_{1},\cdots ,v_{m-1}}\right)$ shows the ratio of $N_{b}\left(\pi_{v_{0},v_{1}^{},\cdots v_{m-1}}\right)$ to $z_{i}^{m,c}$.
(5) According to the definition of Shannon entropy, the FDispEn of original time series x is defined as:
  (8) $$FDispEn\left(x,m,c,d\right)=-\sum_{\pi =1}^{{\left(2c-1\right)}^{m-1}}p\left(\pi_{v_{0},v_{1},\cdots v_{m-1}}\right)In\left(p\left(\pi_{v_{0},v_{1},\cdots v_{m-1}}\right)\right)$$

As an example, let us have a signal x={2,3.5,5.2,4.1,2.2,2.1,2.5,4.6,3.9,7.4}, We set d=1,m=3,c=2, leading to $3^{2}=9$ potential dispersion pattern, ({(−1,−1),(−1,0),(−1,1),(0,−1),(0,0),(0,1),(1,−1),(1,0),(1,1)}). Then, $x_{j}\left(j=1,2,\cdots ,10\right)$ are linearly mapped into two classes with integer indices from 1 to 2 ({1,1,2,1,1,1,1,1,1,2}). Afterwards, a window with length 3 moves along the time series and the differences between adjacent elements are calculated x={(0,1),(1,−1),(−1,0),(0,0),(0,0),(0,0),(0,0),(0,1)}. Afterwards, the number of each dispersion pattern is counted. Finally, using Equation (8) the FDispEn value of x is equal to $-(\frac{1}{8}In\left(\frac{1}{8}\right)+\frac{4}{8}In\left(\frac{4}{8}\right)+\frac{2}{8}In\left(\frac{2}{8}\right)+\frac{1}{8}In\left(\frac{1}{8}\right))=1.2130$.

### 2.3. Performance Analysis of FDispEn

Analyze the impact of different parameters involved in the FDispEn calculation. In the algorithm for Shannon entropy, time delays d in Equation (8) are usually taken about 1, 2, or 3. However, some frequency information may be lost when d>1, so we take d=1 in this paper. For embedding dimensions m, if the embedding dimension m is too small, dynamic changes in the signal may not be detected, and large m may result in small changes in the signal is invisible. We observe that m=2 or 3 is suitable in our study. The parameter c is the number of categories of sequence dispersion in the FDispEn algorithm, when c is too small, data with two large amplitude differences may be assigned to the same classes. Whereas data with a small amplitude difference may be divided into different classes when increasing c. Therefore, this indicates that the FDispEn algorithm is very sensitive to noise. In summary, the parameters we use are d=1, c=3,4,5,6,7 and m=2,3.

**Figure 1:**
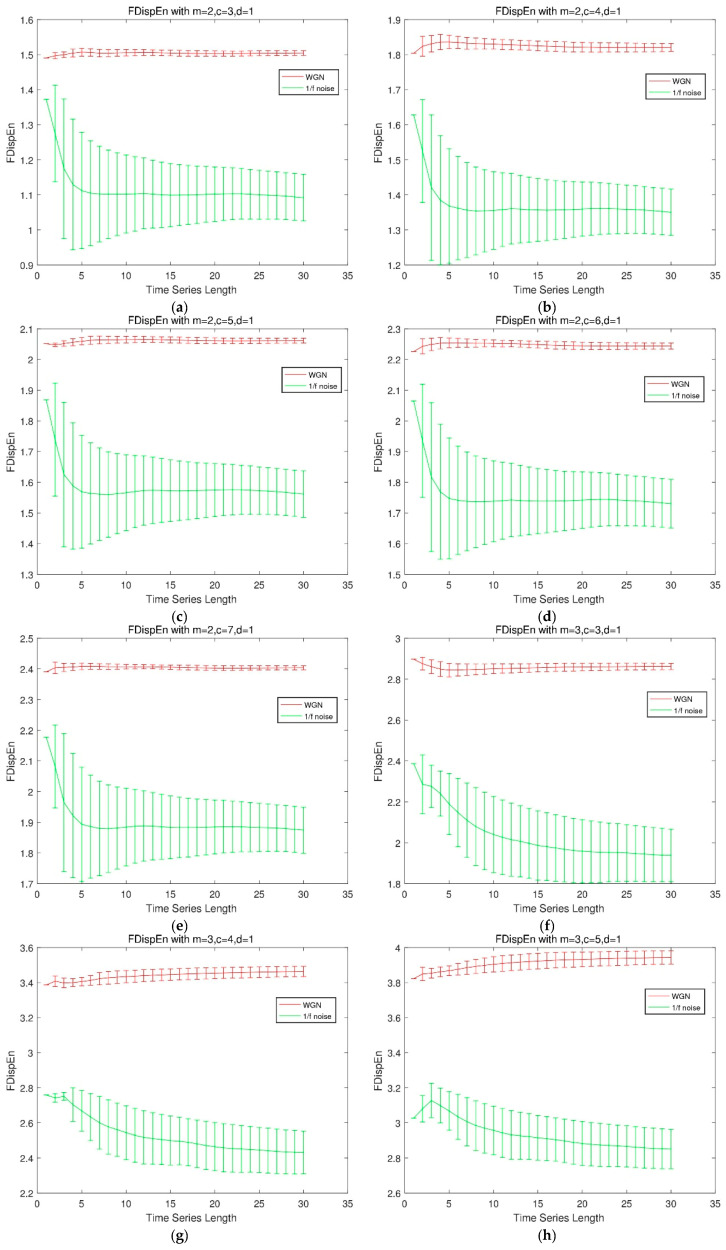

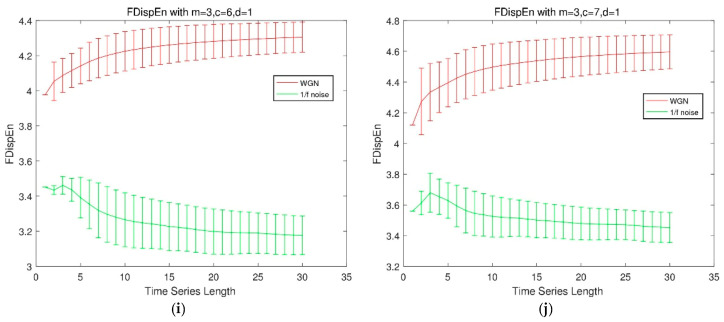
Influence of different parameters on FDispEn.

We compare the FDispEn values of different lengths of Gaussian white noise and 1/f noise under different parameter combinations, and make the mean variance diagram. Gaussian white noise and 1/f noise with size of 3000 are divided into 30 small groups. The results are shown in Figure 1.

The results illustrate that for different combinations of parameters, the overall trend of the FDispEn values of the two noise is the same under different data size, and the FDispEn value increases as c increases. If m=2, c has little effect on the stability of FDispEn. If m=3, the stability of FDispEn value increases slightly with the increase of c.

(2) The influence of data size on the calculation result of FDispEn

Considering the FDispEn values of different data size of Gaussian white noise and 1/f noise, it can be seen from Figure 1 that the FDispEn value of Gaussian white noise is larger than one of 1/f noise, which is consistent with the fact that the irregularity of Gaussian white noise is higher than 1/f noise. It is worth note that small size of data result in instability of FDispEn, whereas, with increasing size of data, the FDispEn values of Gaussian white noise and 1/f noise are becoming steady around a fixed number after the size exceeding 1000. Therefore, we choose the size of samples at least bigger than 1000 empirically in FDispEn calculation.

(3) Compare the computational performance of FDispEn with different parameters.

**Figure 2:**
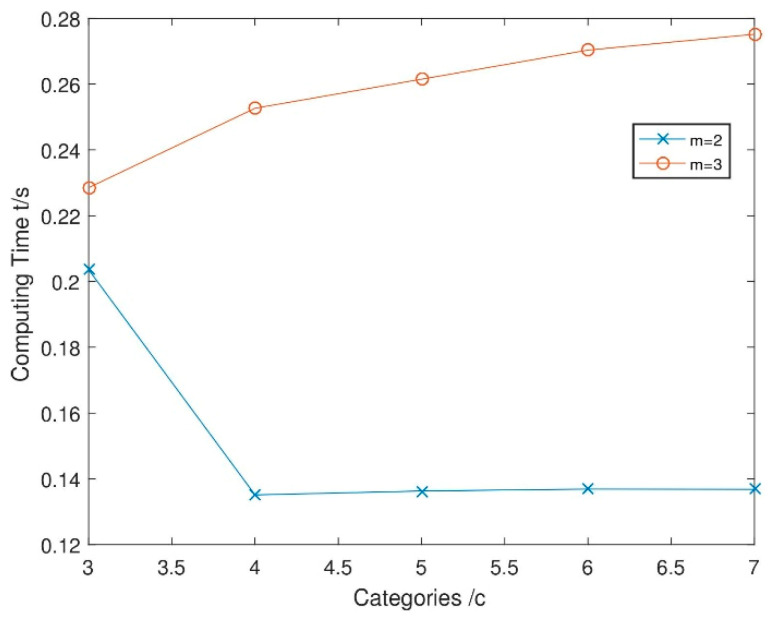
The influence of parameters c and m on calculation time.

This experiment is performed on Windows 7 operating system with Intel Core i7 4-core, 2.5 GHz. and measure the time for data calculation in each small image in Figure 1. The results are shown in Figure 2.

As can be seen from Figure 2, the computational performance degrade with the increase of c and m, which indicates that the smaller c and m should be selected when efficiency is the major consideration. m is selected according to requirements, usually 2 or 3, and ${\left(2c-1\right)}^{m-1}$ should be less than the size of the input ${\left(2c-1\right)}^{m-1}$ is the number of all potential dispersion modes, which is meaningless than the data size). In order to balance the efficiency and accuracy, the parameters we select in this paper are c=3,m=2,d=1.

(4) Comparisons of FDispEn with sample entropy (SampEn) and permutation entropy (PE)

**Figure 3:**
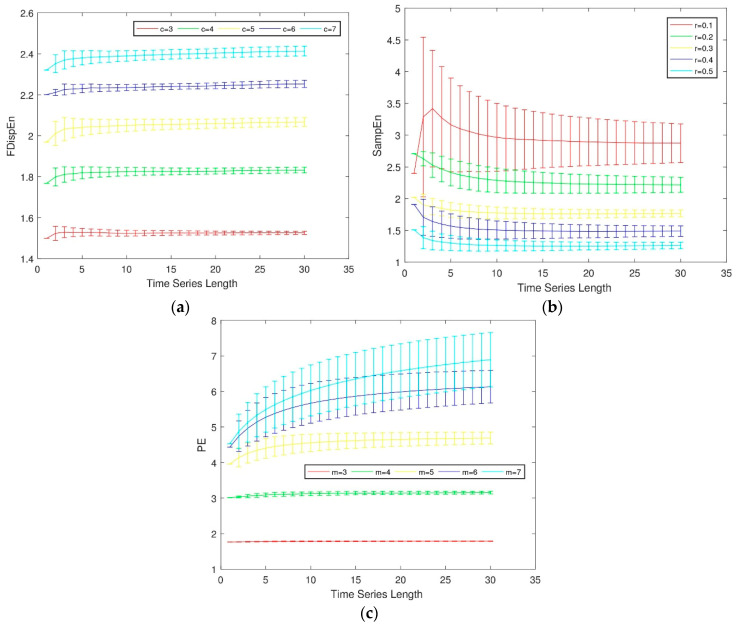
Effect of parameters c, r and m on (**a**) fluctuation-based dispersion entropy, (**b**) sample entropy and (**c**) permutation entropy.

In fact, the concept of similar tolerance r in SampEn is also used in the algorithm of FDispEn. Consider the effect of different size of Gaussian white noise on FDispEn, SampEn and PE. When m=2, the different values of r in SampEn are compared with the values of c in FDispEn, and the results are shown in Figure 3. The data with different magnitudes in the original signal are classified into the same classes or different classes by using FDispEn, which makes FDispEn more robust when dealing with noisy signals than SampEn. The dispersion pattern in FDispEn is similar to the arranging pattern in PE, but these two modes are processed differently. As shown in Figure 3, FDispEn is more stable than PE when processing noise. Since PE only considers the ordered structure of the time series and does not consider the amplitude of the sequence, it leads to the loss of some key information. In summary, compared with SampEn and PE, FDispEn has the advantage of introducing class division and alignment, making it more stable when dealing with noisy signals.

## 3. Feature Extraction Method of Ship-Radiated Noise Using FDispEn and ITD

### 3.1. Feature Extraction Technique Using FDispEn and ITD

**Figure 4:**
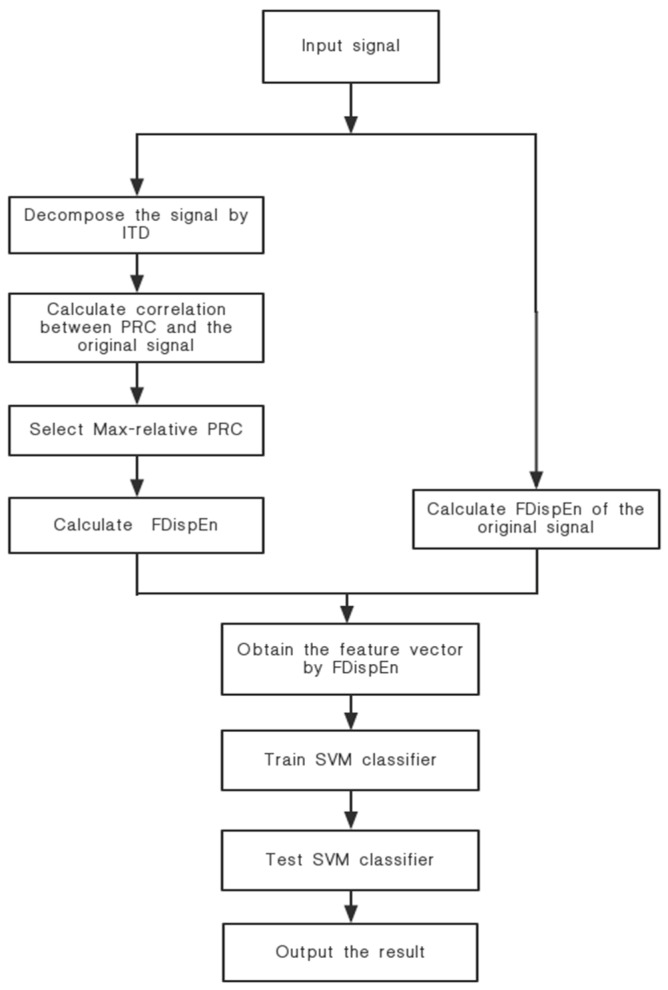
The flowchart of the proposed method.

The basic theories of FDispEn and ITD and their respective advantages have been introduced in Section 2. In this section, we propose a new feature extraction technique using FDispEn and ITD. The detailed flowchart of the proposed method is shown in Figure 4. Specific steps are as follows:

1. Perform ITD decomposition on different types of ship-radiated noise, and decompose to obtain a series of proper rotation components and one residual component.
2. Calculate the correlation between several proper rotation components obtained from the decomposition and the original signal, and select the proper rotation component with large correlation coefficients as the characteristic parameter to calculate the fluctuation-based dispersion entropy value.
3. Compare the fluctuation-based dispersion entropy of ship-radiated noise without ITD decomposition, so as to realize the feature extraction of ship-radiated noise complexity.
4. The result of Step 2 as the feature vector is input into the support vector machine for classification to verify the effectiveness of this method.

### 3.2. Application

**Figure 5:**
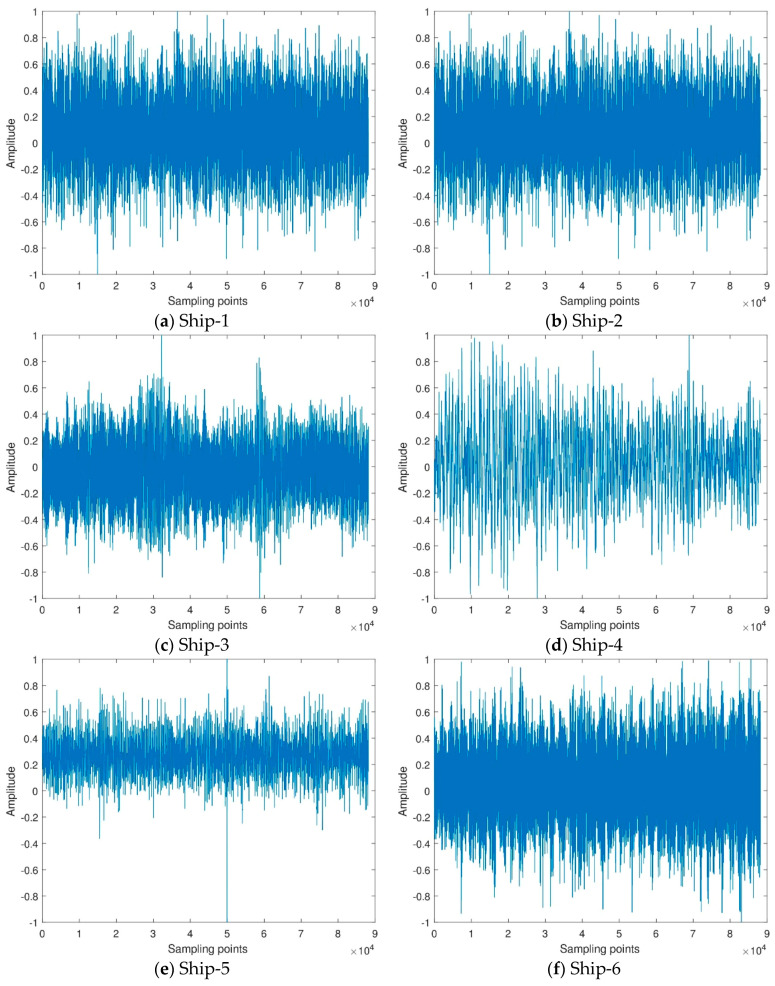

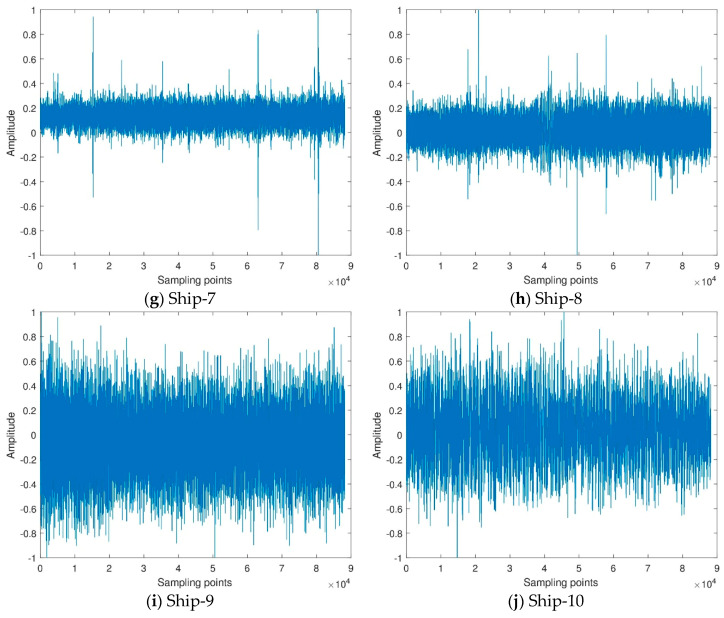
The time-domain waveform of ten types of ship-radiated noise.

In order to demonstrate the effectiveness of the ship-radiated noise complexity feature extraction method based on ITD and fluctuation-based dispersion entropy, this paper uses all data of actual ship-radiated noise measured in a sea area of South China Sea and under the same conditions. Ten different types of ship-radiated noise signals are selected as sample data, namely cruise ship, small diesel ship, whining propeller ship, submarine, etc. For convenience, we respectively named the ten ship-radiated noise as Ship-1, Ship-2, Ship-3, etc. The size of each type of ship-radiated noise is 88200. The sample rate of Ship-1, Ship-2, Ship-3, Ship-4, Ship-9 and Ship-10 are 44.1 kHz. The sample rate of Ship-5, Ship-7 and Ship-8 are 5273 Hz. The sample rate of Ship-6 is 8 kHz. The time domain waveforms of the normalized ship-radiated noise signals are shown in Figure 5.

#### 3.2.1. Analysis of Ship-Radiated Noise Using ITD

**Figure 6:**
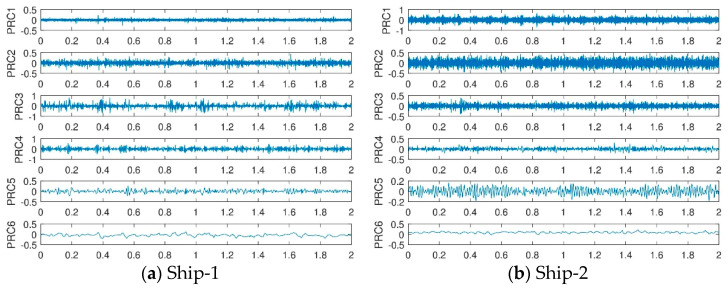

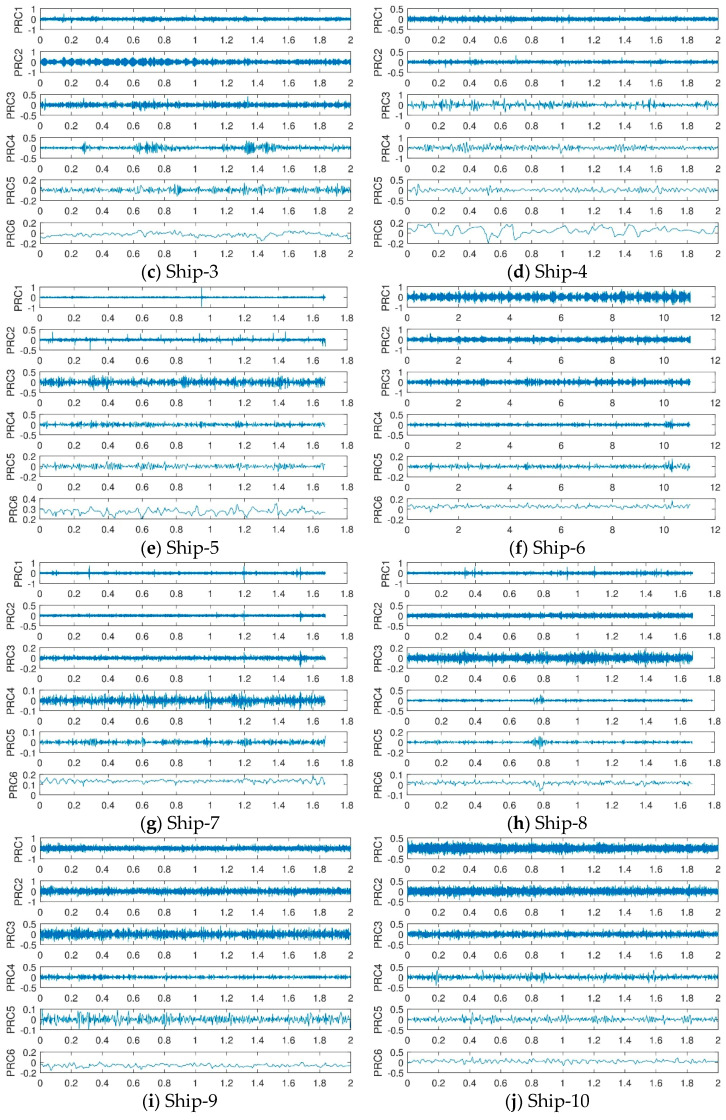
Time domain waveform of results of intrinsic time-scale decomposition (ITD) for ten types of ship-radiated noise signals.

**Figure 7:**
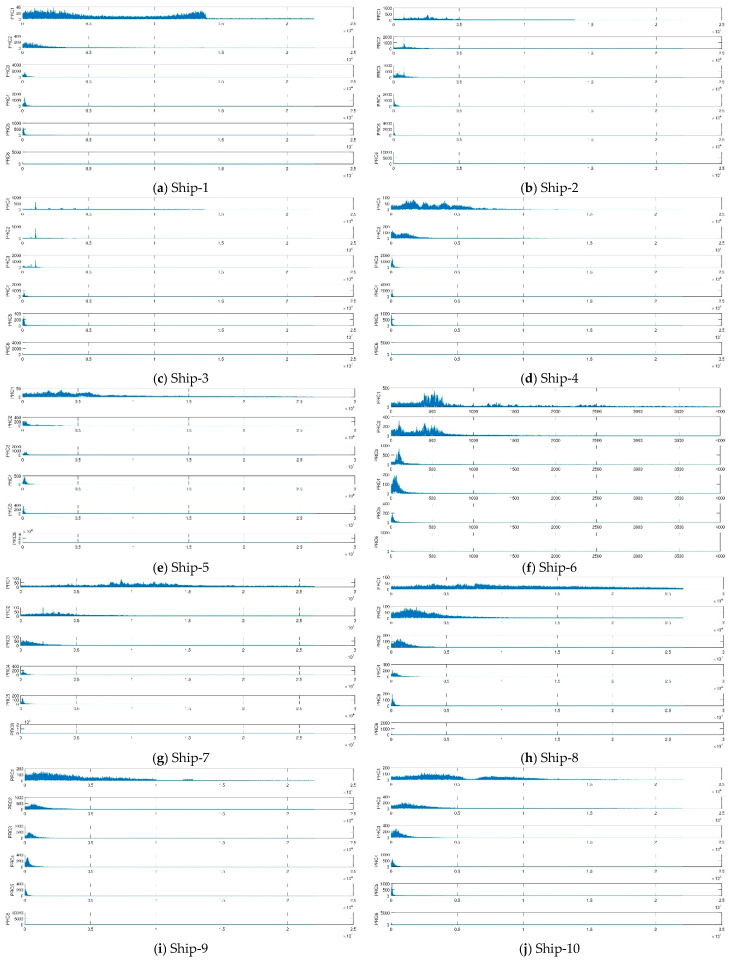
Spectrum of results of ITD for ten types of ship-radiated noise signals.

The time domain waveform and spectrum of ITD decomposition of ten types of ship-radiated noise signals are shown in Figure 6 and Figure 7.

It can be seen from the Figure 6 and Figure 7 that the PRCs components of the ship-radiated noise signals after ITD decomposition are arranged from high frequency to low frequency. The first order mode PRC1 of the ten types of signals indicates the shortest oscillation period of the signal, which is usually the noise component, or the high frequency component of the signal. The orders of ten types of the ship-radiated noise signals depend on the complexity of the signals, in other words, the more complex the signal is, the more PRCs orders are decomposed. Furthermore, we observe that most information characteristics are concentrated in the first PRC component because the amplitudes of these components are much higher than the others.

#### 3.2.2. Fluctuation-Based Dispersion Entropy of Each Order PRC

**Figure 8:**
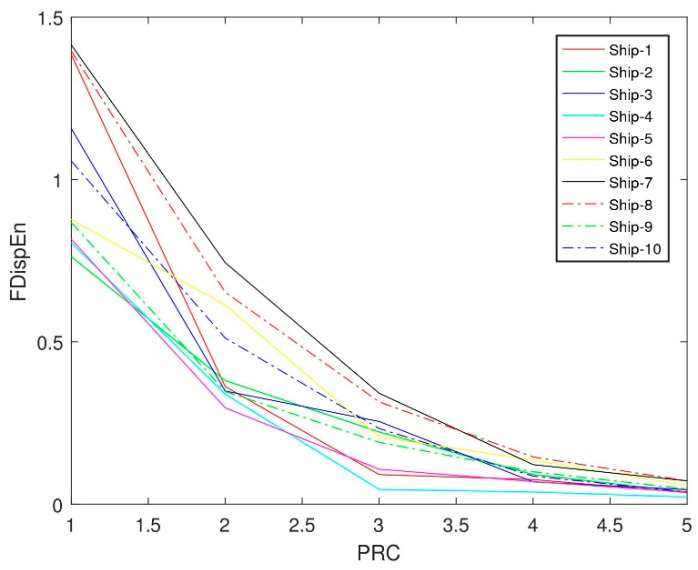
Fluctuation-based dispersion entropy of the proper rotation components (PRCs) of the ten types of ship-radiated noise signals.

After ITD decomposition, the fluctuation-based dispersion entropy of each order PRC of the ten types of ship-radiated noise signals are calculated separately. Figure 8 shows the fluctuation-based dispersion entropy of each order PRC of the ten types of ship-radiated noise signals, and the abscissa is the ten types of ship-radiated noise signals from 1 to 5 order PRC, and the ordinate indicates the fluctuation-based dispersion entropy corresponding to each order PRC. 

#### 3.2.3. The Fluctuation-Based Dispersion Entropy of PRCs with the Highest Correlation Coefficient

The fluctuation-based dispersion entropy of the ten types of ship-radiated noise signals are classified in order of PRCs, which are very different. We select the PRCs characterizing the main information characteristics of the signal, and calculate the differences of the fluctuation-based dispersion entropy to analyze their separability. After the ITD decomposition of the ten types of signals, the PRCs are sorted in descending order in terms of frequency. Usually, because the main information characteristics of the original signals are only concentrated in the first few orders of RPCs, we choose five of them to calculate their correlation. Table 1 lists the correlation coefficients of the various stages of PRCs after the noise mode have been removed from the ten types of ship-radiated noise signals.

The fluctuation-based dispersion entropy of PRCs with the highest correlation coefficient can be defined as the fluctuation-based dispersion entropy by filtering out the PRCs with the highest correlation coefficient. Table 2 lists the fluctuation-based dispersion entropy and distribution of PRCs with the highest correlation coefficient in the ten types of ship-radiated noise signals. It can be seen from Table 2 that the PRCs with the largest correlation between the different ship-radiated noise signals are distributed in different modes, and the values of the fluctuation-based dispersion entropy of PRCs with the highest correlation coefficients have a certain difference.

## 4. Comparison of Feature Extraction Methods of Ship-Radiated Noise 

**Figure 9:**
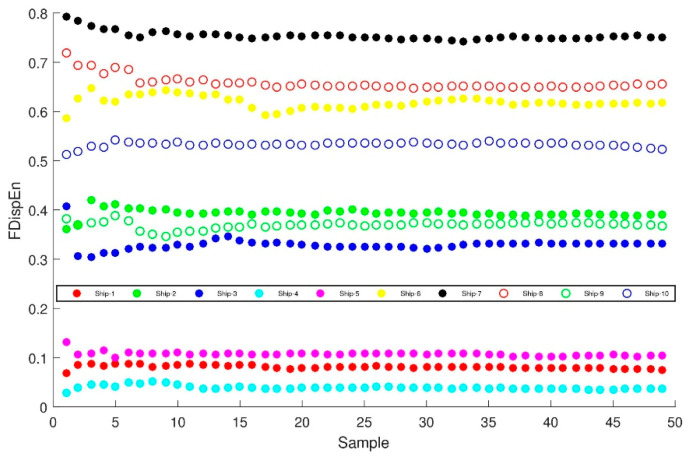
Ten types of ship-radiated noise of fluctuation-based dispersion entropy of the PRC with highest correlation coefficient distribution.

**Figure 10:**
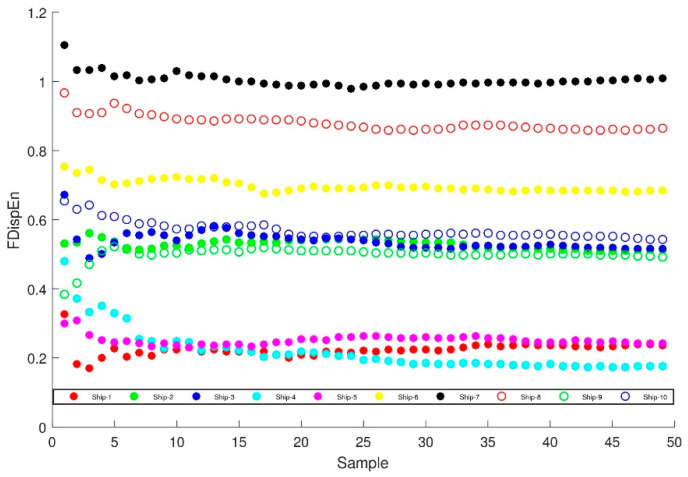
Ten types of ship-radiated noise of fluctuation-based dispersion entropy distribution.

In order to verify the generality of the characteristic parameter, which is the FDispEn of PRC with the highest correlation coefficient, regarded as representing the difference of ten types of signals. There are 50 pieces of sample data of each type of signal randomly selected to calculate the fluctuation-based dispersion entropy and compare with the fluctuation-based dispersion entropy of the original signal without ITD decomposition. In Figure 9, the abscissa is the number of samples, and the ordinate represents the fluctuation-based dispersion entropy of the PRC with the highest correlation coefficient. Figure 10 illustrates the complexity of the dominant PRC of the signal. The PRC of the same ship-radiated noise signal fluctuates within a certain small range, but the value of the ship-radiated noise varies much larger. The above manifest that the proposed feature extraction method can distinguish ten types of ship-radiated noise.

In order to prove the superiority of the proposed method, the fluctuation-based dispersion entropy of original ship-radiated noise is taken as the feature vector of ship-radiated noise. As shown in Figure 10, the fluctuation-based dispersion entropy of Ship-2, Ship-3, Ship-9 and Ship-10 are basically between 0.4 and 0.7. The fluctuation-based dispersion entropy of Ship-1, Ship-4 and Ship-5 are basically between 0.2 and 0.4. Therefore, it is not feasible to distinguish these ships directly by using fluctuation-based dispersion entropy. 

Table 3 provides the fluctuation range, mean and standard deviation of parameters of ten types of ship-radiated noise with 50 samples per type. For convenience, we named the mean of the original signal, the standard deviation of the original signal, the detailed fluctuation range of original signal, the mean values of the fluctuation-based dispersion entropy of PRC with the highest correlation coefficient, the standard deviation values of the fluctuation-based dispersion entropy of PRC with the highest correlation coefficient, and the detailed fluctuation range of the fluctuation-based dispersion entropy of the PRC with the highest correlation coefficient as Mean1, Std1, Range1, Mean2, Std2 and Range2, respectively. It can be concluded from Table 3 that the Mean2 are different and the Range2 are not overlapped, thus the distinguishability of FDispEn of highest correlative PRC makes it very suitable to be used as characteristic parameter while others have very close fluctuation-based dispersion entropy and the ranges of fluctuations are severely overlapping and not non-separable. When the number of samples increases to 100, there is no significant difference in the fluctuation-based dispersion entropy characteristic parameters of the PRC with the highest correlation coefficient, which indicates the corroborates the capability of generalization of this approach. The above results show that the fluctuation-based dispersion entropy of the PRC with the highest correlation coefficient can accurately distinguish different types of ship-radiated noise signals.

## 5. Classification

**Figure 11:**
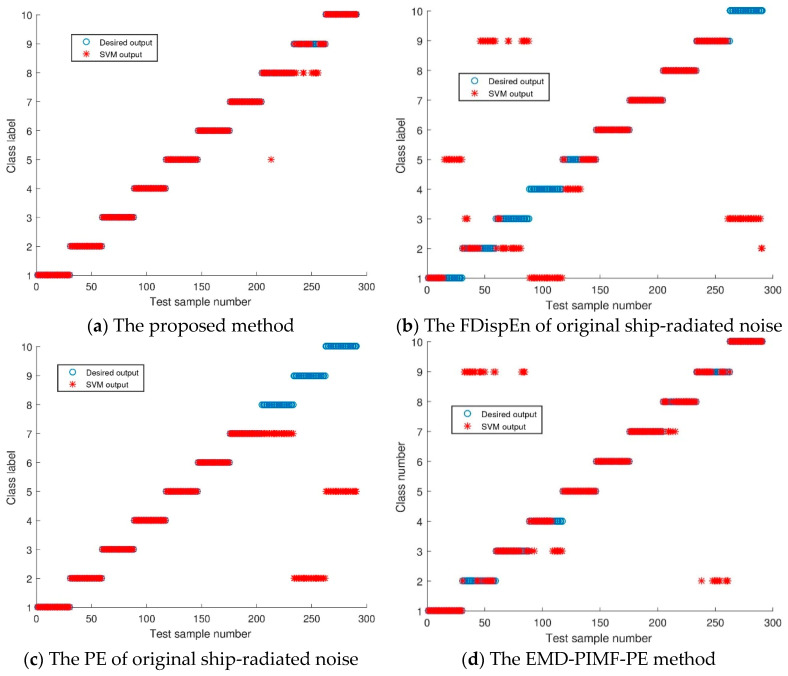
SVM classification results of different methods.

To realize the automatic identification of ship-radiated noise, the extracted features are input into the SVM [21] for training and testing. For each type of ship-radiated noise, 20 samples are used as training samples, and the remaining 30 samples are used as test samples. To compare classification accuracy, FDispEn of the original ship-radiated noise, PE of the original ship-radiated noise, the EMD-PIMF-PE method [22] and ITD-FDispEn are used to classify ship-radiated noise. The SVM outputs of these four methods are shown in Figure 11, respectively, and the recognition rates are listed in Table 4. For each type of ship-radiated noise, the FDispEn of the original signal is not completely classified correctly, and the classification accuracy is 54.2955%. The PE of the original signal method is inferior to the FDispEn of the original signal method, and the classification accuracy is 70.1031%. The EMD-PIMF-PE method is inferior to the PE of the original signals method and classification accuracy is 83.1615%. Compared with the other three methods, the classification accuracy of the proposed method reaches 95.8763%. The results indicate that the proposed method can better classify the ten types of ship-radiated noise.

## 6. Conclusions

A novel feature extraction technique for ship-radiated noise is proposed based on ITD and the fluctuation-based dispersion entropy. The crucial contributions in this paper are highlighted as follows:

1. ITD method as a novel signal decomposition is introduced. ITD method accurately extract the dynamic characteristics of non-stationary signal with fewer numbers of iterations and a small edge effect such that it is capable of online processing in real time. It was first applied to underwater acoustic signal decomposition.
2. Simulation experiments demonstrate that the fluctuation-based dispersion entropy has the advantage of introducing class division and alignment, making it more robust when dealing with noisy signals compared with SampEn and PE. Therefore, this paper applied the fluctuation-based dispersion entropy to underwater acoustic signal processing.
3. Analysis of the separability of the fluctuation-based dispersion entropy of each order PRC, it is often the case that only one PRC with the principal features is selected for feature extraction. In this paper, the entropy is weighted by the highest correlation coefficient, so the importance of each PRC is considered.
4. The method proposed in this paper can extract the characteristics of ship-radiated noise more precisely and comprehensively. The classification recognition rate for ten types of ship-radiated noise signals is 95.8763%.

## Figures and Tables

**Table 1:** Correlation of various orders of proper rotation components (PRCs) for ten types of ship-radiated noise signals.

Order	Ship-1	Ship-2	Ship-3	Ship-4	Ship-5	Ship-6	Ship-7	Ship-8	Ship-9	Ship-10
PRC2	0.4754	0.7200	0.7637	0.3532	0.5062	0.6947	0.5545	0.6679	0.8094	0.5749
PRC3	0.7777	0.5563	0.5489	0.752	0.8388	0.6541	0.5006	0.6401	0.7203	0.5726
PRC4	0.7891	0.3765	0.4537	0.777	0.6489	0.2646	0.5288	0.5063	0.3332	0.5719
PRC5	0.1341	0.3501	0.2130	0.4045	0.2193	0.0176	0.3718	0.3246	0.0482	0.5568
PRC6	0.0303	0.1105	0.0882	0.0589	0.0187	0.0049	0.1372	0.0217	0.0038	0.3191

**Table 2:** Ten types of ship-radiated noise signals of PRC of fluctuation-based dispersion entropy and distribution.

Parameter	Ship-1	Ship-2	Ship-3	Ship-4	Ship-5	Ship-6	Ship-7	Ship-8	Ship-9	Ship-10
The order of PRC with the highest correlation coefficient	PRC4	PRC2	PRC2	PRC4	PRC3	PRC2	PRC2	PRC2	PRC2	PRC2
Fluctuation-based dispersion entropy	0.0759	0.3807	0.3485	0.0376	0.1078	0.6134	0.7440	0.6528	0.3471	0.5117

**Table 3:** Characteristic parameters of ten types of ship-radiated noise signals.

Parameter	Ship-1	Ship-2	Ship-3	Ship-4	Ship-5	Ship-6	Ship-7	Ship-8	Ship-9	Ship-10
Mean1	0.2240	0.5282	0.5374	0.2188	0.2513	0.6972	1.0039	0.8806	0.4996	0.5693
Std1	0.0209	0.0129	0.0276	0.0621	0.0143	0.0175	0.0196	0.0224	0.0227	0.0252
Rang1	0.1693~0.3277	0.5087~0.5620	0.4872~0.6716	0.173~0.4801	0.2314~0.3097	0.6764~0.7538	0.9799~1.1043	0.8576~0.9662	0.382~0.522	0.542~0.6552
Mean2	0.0808	0.3939	0.3294	0.039	0.1072	0.6185	0.7540	0.6583	0.3694	0.5329
Std2	0.0037	0.0085	0.0138	0.004	0.0044	0.0125	0.0094	0.0145	0.0073	0.0051
Range2	0.0687~0.088	0.3621~0.4199	0.3035~0.4078	0.0286~0.0509	0.0995~0.1319	0.5871~0.6468	0.7431~0.7920	0.6482~0.7195	0.3473~0.3894	0.5126~0.5428

**Table 4:** SVM classification results of different methods.

Methods	Accuracy Rate
The proposed method	95.8763%
The FDispEn of original ship-radiated noise	54.2955%
The PE of original ship-radiated noise	70.1031%
The EMD-PIMF-PE method	83.1615%
